# Comparison of the cost-effectiveness of sequential treatment with abaloparatide in US men and women at very high risk of fractures

**DOI:** 10.1007/s40520-023-02682-7

**Published:** 2024-01-30

**Authors:** Mickael Hiligsmann, Stuart L. Silverman, Andrea J. Singer, Leny Pearman, Yamei Wang, John Caminis, Jean-Yves Reginster

**Affiliations:** 1 Department of Health Services Research, CAPHRI Care and Public Health Research Institute, Maastricht University, P.O. Box 616, 6200 MD Maastricht, The Netherlands; 2 Cedars-Sinai Medical Center, Los Angeles and the OMC Clinical Research Center, Beverly Hills, CA USA; 3 MedStar Georgetown University Hospital and Georgetown University Medical Center, Washington, DC USA; 4 Radius Health, Inc., Boston, MA USA; 5 Division of Public Health, Epidemiology and Health Economics, University of Liège, Liège, Belgium; 6 World Health Organization Collaborating Center for Epidemiology of Musculoskeletal Health and Aging, Liège, Belgium

**Keywords:** Abaloparatide, Alendronate, Cost-effectiveness, Gender, Osteoporosis, Sequential

## Abstract

**Background:**

Osteoporotic-related fractures represent an increasing burden to patients, health care systems and society.

**Aims:**

This study estimated cost-effectiveness of sequential treatment with abaloparatide (ABL) followed by alendronate (ALN) compared to relevant alternative strategies in US men and women aged 50 to 80 years at very high fracture risk (bone mineral density T-score ≤  − 2.5 and a recent fracture).

**Methods:**

A lifetime Markov-based microsimulation model was used to estimate healthcare costs and quality-adjusted life years (QALYs). Comparators were sequential treatment with unbranded teriparatide (TPTD)/ALN, generic ALN monotherapy, and no treatment. Analyses were conducted based on initial fracture site (hip, vertebral, or any fracture) and treatment efficacy data (derived from clinical trials or a recent network meta-analysis).

**Results:**

From all analyses completed, sequential ABL/ALN demonstrated more QALYs for lower healthcare costs versus unbranded TPTD/ALN. No treatment was dominated (higher costs for less QALYs) versus ALN monotherapy. Sequential ABL/ALN resulted in favorable cost-effectiveness (at US threshold of $150,000/QALY) versus generic ALN monotherapy in men aged ≥ 50 years with any fracture type, women aged ≥ 65 years with any fracture type, and women aged ≥ 55 years having a hip or vertebral fracture.

**Discussion:**

Similar cost-effectiveness of sequential ABL/ALN versus unbranded TPTD/ALN, ALN monotherapy, and no treatment was observed in both US men and women at very high fracture risk, with a moderate improvement in cost-effectiveness in men versus women and in patients with a hip or vertebral fracture.

**Conclusions:**

Sequential therapy with ABL/ALN was cost-effective in US men and women at very high risk of fractures.

**Supplementary Information:**

The online version contains supplementary material available at 10.1007/s40520-023-02682-7.

## Introduction

Osteoporotic-related fractures represent a massive and increasing burden on patients, healthcare systems, policymakers, and society. It is estimated that one out of four men and one out of two women aged 50 years will have an osteoporotic fracture during their remaining lifetime [1, 2]. Fractures, especially at the hip or spine, are associated with increased morbidity, mortality excess, and have a significant impact on quality of life. In 2016, 2.1 million osteoporotic fractures occurred among US Medicare patients: 25% at the spine and 17% at the hip [3]. In the 27 countries of the European Union as well as the United Kingdom and Switzerland, the number of fragility fractures in 2019 was estimated at 4.3 million in people aged ≥ 50 years, of which about 30% occurred in men [4]. The total economic burden of these fractures was estimated at €57 billion. With increasing life expectancy, the number of fractures is anticipated to increase by 25% in the next 15 years, and even more so in men [4]. The substantial and increasing burden of osteoporosis in men has revealed the critical need to identify and manage what was thought to be a disease primarily of women.

It is further recognized that the risk of subsequent fractures increases significantly after an initial fracture [5]. Patients with at least one previous fragility fracture with a diagnosis of osteoporosis are considered at very high risk of subsequent fractures [6–8]. Despite this population being most likely to sustain a new fracture, a vast majority is not receiving an osteoporosis medication [4, 9]. Recently, an expert working group [8] has recommended the use of sequential treatment for patients found to be at very high risk, beginning with an anabolic and followed by maintenance therapy using an antiresorptive agent, in line with clinical studies showing a better risk reduction with sequential treatment compared to an antiresorptive agent alone [10, 11]. Sequential therapies, however, are more expensive, and economic evaluations are therefore increasingly important to inform decision makers about the potential economic value of this strategy [12]. A recent systematic review of cost-effectiveness analyses of sequential therapies published with data to June 2022 [13] identified a few studies that suggested the cost-effectiveness of sequential treatment with either abaloparatide (ABL) or romosozumab in populations at very high risk. All the studies included in this review were, however, conducted in postmenopausal women with osteoporosis. Another recent systematic review of cost-effectiveness studies conducted in men with osteoporosis [14] found that economic evaluations in men are lacking compared to studies in women and that there is limited information on the comparability of the cost-effectiveness of drugs between men and women.

Recently, we showed the cost-effectiveness of sequential therapy with ABL followed by alendronate (ALN) in US men at high risk of fracture [12]. Although results were rather similar to what was observed in postmenopausal women with osteoporosis [15, 16], it is difficult to make a direct comparison of studies between men and women, as several model parameters are different, including various populations, fracture risk, fracture costs and different model assumptions such as adherence scenarios, time-dependent risk of subsequent fractures, or drug prices. A direct comparison using a systematic approach would reveal whether the cost-effectiveness of sequential ABL/ALN is similar in both men and women at very high risk of fractures. Integrating gender into cost-effectiveness analyses is necessary to build rigorous evidence to capture a more accurate picture of the economic impact of sequential therapy, with potential implications on healthcare decision-making and health inequalities between genders in particular. This study was therefore designed to assess and compare the cost-effectiveness of sequential treatment with ABL followed by ALN to alternative strategies in US men and women at very high fracture risk.

## Methods

### Interventions

This study compared lifetime healthcare costs and health outcomes expressed as quality-adjusted life years (QALYs) of sequential ABL/ALN compared to sequential unbranded teriparatide (TPTD)/ALN, generic ALN monotherapy, and no treatment. No treatment is included as a comparator as many patients at very high risk of fracture are not receiving a medication for osteoporosis. In line with clinical practices [6], patients received 18 months of ABL or unbranded TPTD followed by an additional 5 years of ALN. A treatment duration of 5 years was also used for ALN monotherapy. As medication adherence is an important driver of the cost-effectiveness of osteoporosis medications [17], it was included in the model.

### Model structure

A Markov-based microsimulation model was implemented using TreeAge Pro 2023 R1.0 (TreeAge Pro Inc., Williamston, MA, USA), and was similar to the model used recently in Hiligsmann et al. [12]. All costs were adjusted for inflation by the US consumer price index for medical care to 2022 US dollars, and were discounted, as QALYs, annually by 3% [14]. The model consisted of the following health states: “high risk,” “hip fracture,” “vertebral fracture,” “nonhip nonvertebral fractures (NHNV)” and “death” (Online Resource 1). All patients begin in the “high risk” health state where the patient was a 70-year-old man or woman with a bone mineral density (BMD) T-score ≤  − 2.5 and a recent fracture, in line with definitions of very high risk in the US [6, 7]. Patients moved between health states in the model according to transition probabilities, and costs and health outcomes (life years and health utility) were captured for all individuals during all cycles. A total of 1,000,000 individual patients were simulated for every analysis to guarantee the stability of the results. Each cycle was set to 6 months and patients could have multiple fractures during their lifetime at different fracture sites. Analyses were conducted from the US healthcare decision maker perspective [18]. A similar structure of the model was used for both men and women, while gender-specific data (derived preferably from the same references) were used whenever possible. Key model inputs and assumptions are described below (and in Table 1), while additional information on the model is available in Hiligsmann et al. [12].

**Table 1:**
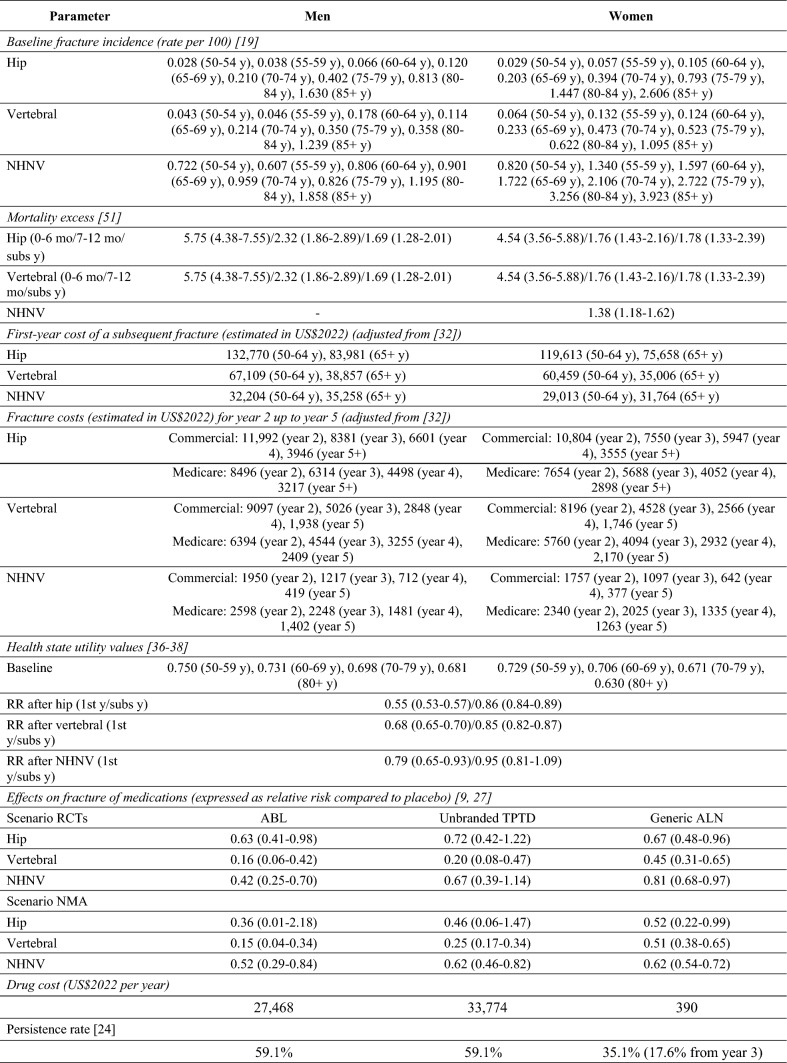
Model data

*ABL* abaloparatide*, ALN* alendronate*, NHNV* nonhip nonvertebral*, NMA* network meta-analysis*, RCT* randomized controlled trial*, RR* relative reduction*, subs* subsequent*, TPTD* teriparatide

### Transition probabilities

The baseline age- and gender-specific risk of fractures used in the model combined the general population fracture risk and increased risks associated with osteoporosis (BMD T-score ≤  − 2.5) and with a recent fracture. The fracture incidences in the US general population were extracted from Ettinger et al. [19], in line with the current US FRAX^®^ Tool and recent published economic studies [12, 20]. A commonly used method [21] was applied to derive the increased risk associated with osteoporosis, using the US Caucasian female BMD reference database to derive T-scores in both men and women [22]. Time-dependent (6-month intervals) relative risks of subsequent fractures were used for patients with at least one fracture [5] and were higher for men than women [23].

During simulation, fracture risk was updated when the patient age changed and after a new fracture occurred. In case of multiple previous fractures, only one (the highest) increased risk was used in the model. A relative fracture risk reduction was further applied during the treatment period and during a posttreatment period (called offset time) where the treatment effect was declining. Treatment persistence was modeled according to the methodology of Liu et al. [24] and using persistence levels from the US study of Cheng et al. [25].

Two scenarios for the treatment effects were investigated: (1) efficacy data from randomized controlled trials (RCTs) and (2) efficacy data from a network meta-analysis (NMA). The first scenario used similar data and assumptions as Hiligsmann et al. [12]. Therefore, fracture risk reduction for ABL and unbranded TPTD were derived from the 43-month ACTIVE/ACTIVExtend Trial [10] conducted in postmenopausal women with osteoporosis, and the effect of ALN on fracture risk, used in both sequential and monotherapy strategies, was derived from the National Institute for Health and Care Excellence (NICE) appraisal (TA464) [26]. The second scenario was based on the recent study of Willems et al. [27] that conducted an NMA of all RCTs of osteoporosis medications for postmenopausal women with osteoporosis up to September 2020. The fracture risk reductions of TPTD and ABL at 24 months were used in our model, while fracture risk reduction at 36 months was used for ALN. Due to similar gains of osteoporosis medications on BMD in men and women [28, 29] and the lack of fracture efficacy data in men, similar treatment efficacy (derived from studies with postmenopausal women with osteoporosis) was used for both men and women.

Age- and gender-specific mortality rates (in 2019) were derived from US national statistics. Mortality after hip and vertebral fractures was incorporated in the model, consistent with prior economic studies [12]. Mortality after NHNV fractures was included for women but not for men due to the lack of significant effect [30]. In line with the International Osteoporosis Foundation–European Society for Clinical and Economic Aspects of Osteoporosis and Osteoarthritis (IOF-ESCEO) guideline for economic evaluations in osteoporosis [31], 25% of the fracture excess death was considered to be attributable to fractures.

### Costs

As the model was developed from the US payer decision maker perspective, only the direct medical care costs, including drug acquisition, monitoring, management of adverse events, fracture hospitalization, or rehabilitation were considered. Yearly incremental medical costs of hip, vertebral, and NHNV fractures for Medicare- and commercially insured women were derived from Tran et al. [32] and were adjusted to reflect higher costs of a second fracture [33]. Costs in subsequent years up to five years after an initial fracture from the same study [32] were also included in the model. As hip fractures are associated with long-term admission to nursing home and high associated costs [31], the incremental cost of hip fractures in year 5 was maintained for lifetime. In cases of multiple fractures, only one (the highest) fracture cost was considered. As men experienced higher fracture costs than women, all fracture costs were increased by 11% in men, as suggested by Williams et al. [34].

Drug prices were derived from the wholesale acquisition cost (WAC) price from the online Red Book in 2022. Yearly cost of ABL, unbranded TPTD, and generic ALN were thus US$27,468, US$33,774, and US$390, respectively. Total drug costs were adjusted by number of drugs taken during the ACTIVE trial [29] to allow for the fact that patients did not receive all drugs. Monitoring costs included one physician visit of (US$118) every six months and one BMD measurement at a cost of US$47.50 every two years in line with Medicare insurance reimbursement. We also considered the costs associated with managing treatment adverse events, as done previously [12].

### Health utility

Health benefits were expressed in QALYs measuring the impact of treatments on quantity and quality of life. To generate QALY, health utility summarizing quality of life between 0 (corresponding to death) and 1 (corresponding to perfect health) is needed. Baseline age- and gender-specific utility was derived from the report of nationally representative values for the noninstitutionalized US adult population (2006 data using EQ-5D) [35] and were reduced by 13% to reflect the lower utility of US patients with fracture compared to the general population [36]. The effects of new fractures on utility were derived from the large international ICUROS study [37] and from Kanis et al. [38] for NHNV fractures. Similar fracture effect on utility was assumed for men and women, in line with a recent study suggesting that men and women had a similar quality of life one year after fracture [39].

### Base-case and sensitivity analyses

Four base-case analyses were conducted in patients aged 70 years according to gender (men and women) and the two treatment efficacy data scenarios (RCT efficacy data, NMA efficacy data). An intervention is dominated if it provides less QALYs for more costs than another intervention. Incremental cost-effectiveness ratios (ICERs), defined as the difference between two strategies in terms of total healthcare costs divided by their difference in QALYs, were estimated. If the ICER is below the cost-effectiveness threshold representing decision makers’ willingness to pay, the intervention is considered cost-effective. In the US, a threshold of US$150,000 per QALY gained has been recommended for interventions that offer considerable other benefits [40].

To evaluate the robustness of base-case results and determine key drivers of cost-effectiveness, one-way sensitivity analyses were conducted on age (from 50 to 80 years), fracture incidence (± 25%), fracture costs (± 25%), fracture effects on utilities (± 25%), discount rates (0%, 5%), no fracture excess mortality, ABL drug price (± 20% and 50%), and the offset time of treatment effect (a linear decrease up to three years following discontinuation and a maintenance of the effects two years following discontinuation followed by a linear decline in the following three years). Finally, complete medication adherence was also assessed. One-way sensitivity analyses were presented as tornado diagrams for the four base-case analyses.

To better understand the joint uncertainty of our analyses, probabilistic sensitivity analyses were also done, varying key parameters from specified distributions (see Online Resource 2). Two hundred second-order simulations of 50,000 individual patients were performed and were presented as cost-effectiveness acceptability curves that show the probability of each intervention being cost-effective according to decision makers’ willingness to pay per QALY gained.

The ESCEO-IOF guideline for economic evaluation in the field of osteoporosis [31] and the Consolidated Health Economic Evaluation Reporting Standards (CHEERS) 2022 statement [41] were followed to make sure all relevant components of this economic study were adequately designed and reported appropriately. The completed checklists of items of these guidelines are included in Online Resource 3. The model has been extensively validated and used in the past. For the purpose of this study, US clinical experts were involved in the design of the health economic plan and approved the final version with all data and assumptions. Validation efforts included running the model with other parameters and assumptions and comparison of predicted outcomes (fractures, life expectancies) with other published studies.

## Results

### Base-case analyses

In the four base-case analyses (Table 2), sequential ABL/ALN was associated with an incremental gain of QALY relative to no treatment (ranging from 0.123 to 0.169), to sequential unbranded TPTD/ALN (0.020–0.030), and to ALN monotherapy (0.099–0.121). However, total healthcare costs were higher for sequential ABL/ALN compared to no treatment (US$3944-US$9577) and to ALN monotherapy (US$7389-US$11,226), resulting in ICERs of US$20,378 to US$77,547 per QALY gained of sequential ABL/ALN compared to no treatment, and of US$60,810 to US$113,244 compared to ALN monotherapy. As such, the base-case analyses concluded that sequential ABL/ALN is cost-effective compared to no treatment and to generic ALN monotherapy. Furthermore, sequential ABL/ALN dominated sequential unbranded TPTD/ALN with more QALYs for less costs (US$ − 9211 to US$ − 7621). No treatment was also dominated (higher costs for less QALYs) compared to ALN monotherapy. The ICERs of sequential ABL/ALN were lower in men compared to women and when using NMA efficacy data.

**Table 2:** Lifetime costs (US$), QALYs, fractures, and cost-effectiveness of sequential ABL/ALN compared to alternative treatments in men and women aged 70 years with a recent fracture and BMD T-score ≤  − 2.5 according to treatment efficacy scenarios

	ABL/ALN	No treatment	Unbranded TPTD/ALN	ALN monotherapy
Men using clinical trials efficacy				
Total costs	89,371	82,900	98,582	80,383
QALYs	7.329	7.186	7.302	7.222
Fractures	1.441	1.694	1.507	1.645
ICER		45,246	ABL/ALN Dominant^a^	84,070
Men using NMA efficacy				
Total costs	86,093	82,649	94,283	78,704
QALYs	7.354	7.185	7.331	7.233
Fractures	1.416	1.689	1.453	1.616
ICER		20,378	ABL/ALN Dominant^a^	60,810
Women using clinical trials efficacy				
Total costs	81,007	71,430	89,540	69,781
QALYs	7.804	7.681	7.774	7.705
Fractures	1.610	1.844	1.673	1.802
ICER		77,547	ABL/ALN Dominant^a^	113,244
Women using NMA efficacy				
Total costs	79,253	71,414	86,874	68,743
QALYs	7.822	7.682	7.802	7.716
Fractures	1.598	1.844	1.632	1.779
ICER		56,028	ABL/ALN Dominant^a^	99,362

*ABL* abaloparatide*, ALN* alendronate*, BMD* bone mineral density*, ICER* incremental cost-effectiveness ratio*, NMA* network meta-analysis, *QALY* quality-adjusted life year*, TPTD* teriparatide

^a^Dominant = more QALYs for less costs

Between the two non-dominated interventions, in patients with any recent fracture, sequential ABL/ALN was cost-effective (at the US cost-effectiveness threshold) compared to generic ALN monotherapy in men aged ≥ 50 years and in women aged ≥ 65 years (Table 3). The costs per QALY gained decreased with increasing age and were lower in patients with a hip or a vertebral fracture, leading to the cost-effectiveness of sequential ABL/ALN in women aged ≥ 55 years with a hip or vertebral fracture. Moreover, sequential ABL/ALN was even dominant (more QALYs for less costs) compared to ALN monotherapy in men aged ≥ 75 years with a vertebral fracture and those aged ≥ 70 years with hip fractures using NMA efficacy data. Online Resource 4 Tables S1–6 present the ICERs of sequential ABL/ALN compared to all strategies according to fracture site and treatment efficacy data.

**Table 3:** Cost effectiveness of ABL/ALN vs ALN monotherapy in US men and women with a recent fracture and BMD T-score ≤  − 2.5 according to site of fractures and treatment efficacy scenarios

	Men		Women	
	Clinical trials efficacy	NMA efficacy	Clinical trials efficacy	NMA efficacy
Any recent fracture				
50 years	139,530	145,537	253,015	257,286
55 years	146,804	124,577	166,039	177,166
60 years	94,568	85,629	184,376	187,469
65 years	105,453	83,481	138,395	134,682
70 years	84,070	60,810	113,244	99,362
75 years	67,686	32,751	92,140	73,752
80 years	68,713	29,272	98,505	64,421
Recent hip fracture				
50 years	91,879	88,073	186,967	189,815
55 years	85,588	71,133	110,795	116,045
60 years	48,134	40,002	117,972	124,867
65 years	58,800	43,956	90,609	86,007
70 years	38,542	18,437	69,769	58,738
75 years	23,705	Dominant	48,513	28,148
80 years	23,331	Dominant	46,341	17,990
Recent vertebral fracture				
50 years	62,210	58,547	200,423	219,670
55 years	55,182	43,643	124,909	131,741
60 years	23,792	14,746	138,029	139,889
65 years	30,122	17,231	109,852	105,990
70 years	12,347	Dominant	84,256	71,186
75 years	Dominant	Dominant	62,650	42,951
80 years	Dominant	Dominant	62,381	33,532

*ABL* abaloparatide*, ALN* alendronate*, BMD* bone mineral density*, NMA* network meta-analysis

### One-way sensitivity analyses

**Fig. 1:**
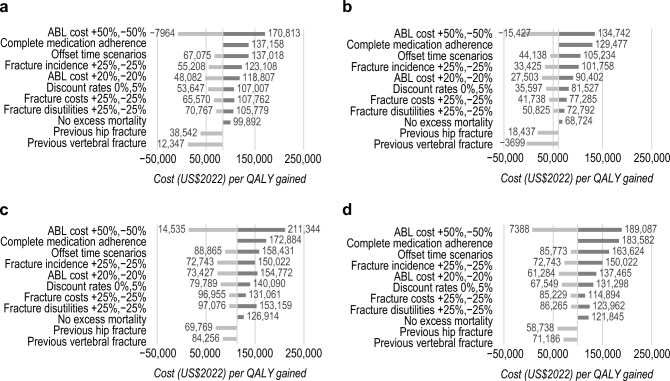
One-way sensitivity analyses on the cost per QALY gained of ABL/ALN compared to ALN monotherapy in **a** men using clinical trials efficacy data, **b** men using NMA efficacy data, **c** women using clinical trials efficacy data and **d** women using NMA efficacy data. *ABL* abaloparatide*, ALN* alendronate*, NMA* network meta-analysis, *QALY* quality-adjusted life year

Base-case analyses were robust over one-way sensitivity analyses that are summarized as tornado diagrams in Fig. 1. Cost of ABL, fracture incidence, offset time, and the site of previous fracture were key model drivers. Assuming complete medication adherence also led to a higher ICER of sequential ABL/ALN compared to ALN monotherapy. In men, sequential ABL/ALN was cost-effective compared to ALN monotherapy in all sensitivity analyses except when assuming a 50% higher drug cost in the RCT’s efficacy data scenario. In women, there were some sensitivity analyses, especially using RCT’s efficacy data, that led to ICERs (slightly) higher than $150,000, in particular when assuming lower drug costs, shorter offset time, or complete medication adherence. As shown in Online Resource 4 Table S7, sequential ABL/ALN remained dominant (more QALYs for less costs) compared to sequential unbranded TPTD/ALN in all sensitivity analyses, except when assuming a 50% higher drug cost of ABL. In that simulation, ABL/ALN led to more costs and QALYs, resulting in ICERs between US$36,082 and US$175,441 per QALY gained. All sensitivity analyses on the cost-effectiveness of sequential ABL/ALN compared to no treatment were below the US cost-effectiveness threshold, except again when ABL price was 50% higher. No treatment was further dominated (less QALY, more costs) compared to ALN monotherapy in all sensitivity analyses.

### Probabilistic sensitivity analyses

**Fig. 2:**
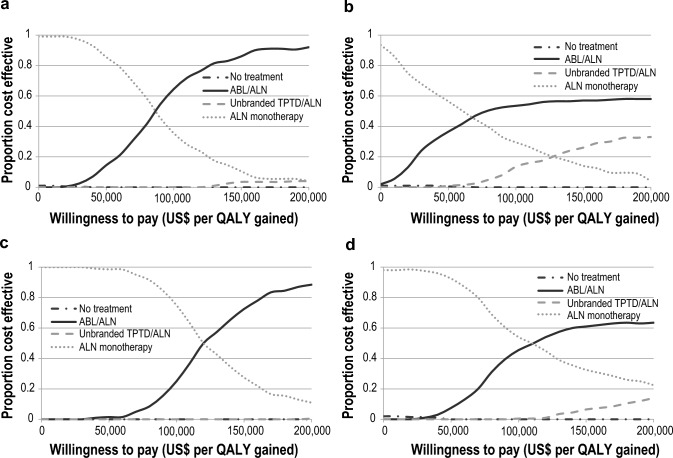
Cost-effectiveness acceptability curves in patients aged 70 years with a recent fracture and BMD T-score ≤  − 2.5, in **a** men using clinical trials efficacy data, **b** men using NMA efficacy data, **c** women using clinical trials efficacy data and **d** women using NMA efficacy data. *ABL* abaloparatide*, ALN* alendronate*, BMD* bone mineral density, *NMA* network meta-analysis, *QALY* quality-adjusted life year*, TPTD* teriparatide

The probabilistic sensitivity analyses confirmed that sequential ABL/ALN was the most cost-effective intervention at the US cost-effectiveness threshold of US$150,000 per QALY gained with probabilities to be cost-effective of 86% (in men using RCT efficacy data), 57% (in men using NMA efficacy data), 73% (in women using RCT efficacy data), and 61% (in women using NMA efficacy data) (see Fig. 2). Online Resource 4 Figures S1–2 show the cost-effectiveness acceptability curves in patients with a recent hip or vertebral fracture, respectively. Online Resource 4 Figure S3 shows the probabilities of a cost-effective outcome for sequential ABL/ALN compared to ALN monotherapy and revealed higher uncertainty when using the NMA efficacy data scenario (resulting from the large confidence interval of the effect of ABL on hip fractures).

## Discussion

Sequential therapy with ABL/ALN was overall cost-effective in US men and postmenopausal women at very high risk of fractures. In all analyses completed, sequential ABL/ALN was associated with more QALYs for less costs compared with sequential unbranded TPTD/ALN, while no treatment was dominated (less QALYs for more costs) by ALN monotherapy. Among the two nondominated interventions, sequential ABL/ALN was cost-effective compared to ALN monotherapy (at the US cost-effectiveness threshold) in men aged ≥ 50 years, in women aged ≥ 65 years with any fracture, and women aged ≥ 55 years with a hip or vertebral fracture.

Generally, for similar age and initial fracture site, men were associated with slightly lower ICERs than women. This result is in contrast with the review of Li et al. [14] that suggested higher ICERs in men in 75% of studies. This finding could however be explained by the very high risk of our population. Indeed, the increased risks due to osteoporosis and to recent fracture were both higher for men compared to women, leading to a greater absolute fracture risk for men at very high fracture risk. Furthermore, consequences of fractures (such as excess mortality or fracture costs) were higher in men. Improved cost-effectiveness was also observed in patients with a hip or vertebral fracture, resulting from the higher risk of subsequent fractures in these patients. In women, the minimum age at which sequential treatment ABL/ALN was cost-effective compared to ALN monotherapy decreased from 65 to 55 years with these fracture types. Furthermore, the ICERs were generally lower when using the NMA efficacy data, due to higher treatment fracture risk reduction in this scenario. However, the age at which sequential ABL/ALN was cost-effective remained similar, suggesting that our conclusions are robust over treatment efficacy scenarios. The limited impact of gender on the cost-effectiveness of sequential therapy therefore does not support different osteoporosis treatment and management strategies in men and women at very high risk of fractures.

This study confirms the economic benefits of treating patients at very high risk of fractures with sequential treatment [8]. Currently, many patients at high fracture risk do not receive an osteoporosis medication [9], and adherence to osteoporosis medication remains suboptimal [42]. As patients with a recent fracture are the most likely to sustain further fractures, it is important to optimize secondary fracture prevention. In particular, fracture liaison services are essential and have been shown to be effective in reducing subsequent fractures [43] and to be cost-effective in combination with oral bisphosphates [44]. Potentially higher economic benefits of fracture liaison services could even be reached when combined with sequential therapy with ABL.

There are potential limitations of this study, of which some were already reported in previous studies [12, 15, 16]. First, a direct comparison of the cost-effectiveness between men and women could be limited by the lack of fracture risk studies in men. In line with regulators accepting a bridging study with a placebo for approval in men, similar treatment efficacy is commonly assumed between men and women with osteoporosis [14, 45]. Second, certain relevant detailed data (such as fracture costs or increased risks of subsequent fracture after fractures) were only available for women, and adjustments needed to be done to consider expected differences between men and women. Another example is the use of similar medication adherence for men and women, while other studies have suggested that men are generally less adherent to osteoporosis medications than women [46]. More gender-specific data on real-world persistence to sequential ABL/ALN would thus be of interest to confirm our findings. Similarly, real-world effectiveness data [47] could be used in future economic evaluations and improve the robustness of our conclusions. Third, the study was conducted using populations considered white or Caucasian men and women. It is nowadays recognized that there are racial and ethnic differences in fracture risk [48] and fracture outcomes [49]. More economic studies are needed to investigate the transferability of our findings to “non-white” US men and women. Finally, this study was limited to QALY as health outcome. Although QALY is the academic standard for measuring health outcomes in economic evaluations, the Institute for Clinical and Economic Review (ICER) introduced in 2018, to supplement, QALY the equal value of life years gained (evLYG) metric which evenly measures any gains in length of life, regardless of the treatment’s ability to improve patients’ quality of life [50]. As fracture prevention, especially in the oldest patients, leads to life extension, high expected benefits are also anticipated with evLYG.

In conclusion, this study suggests similar cost-effectiveness of sequential ABL/ALN compared to unbranded TPTD/ALN, ALN monotherapy, and no treatment in both US men and women at very high fracture risk, with a moderate improvement in cost-effectiveness in men compared to women and in patients with a hip or vertebral fracture.

### Supplementary Information

Below is the link to the electronic supplementary material.

Supplementary file1 (DOCX 414 KB)

Supplementary file2 (DOCX 25 KB)

Supplementary file3 (DOCX 31 KB)

Supplementary file4 (DOCX 83 KB)

## Data Availability

Data that underlie the results reported in a published article may be requested for further research 6 months after completion of FDA or EMA regulatory review of a marketing application (if applicable) or 18 months after trial completion (whichever is latest). Radius will review requests individually to determine whether (i) the requests are legitimate and relevant and meet sound scientific research principles, and (ii) are within the scope of the participants’ informed consent. Prior to making data available, requestors will be required to agree in writing to certain obligations, including without limitation, compliance with applicable privacy and other laws and regulations. Proposals should be directed to info@radiuspharm.com.
