# Supplementary material for: Comparison of the cost-effectiveness of sequential treatment with abaloparatide in US men and women at very high risk of fractures
Source: Aging Clin Exp Res. 2024 Jan 30;36(1):14. doi: 10.1007/s40520-023-02682-7 (PMC10827834; doi:10.1007/s40520-023-02682-7)
Supplement: Supplementary file 1 — Supplementary file1 (DOCX 414 KB) [file 40520_2023_2682_MOESM1_ESM.docx]

**Online Resource 1:** Health economics analysis and model structure


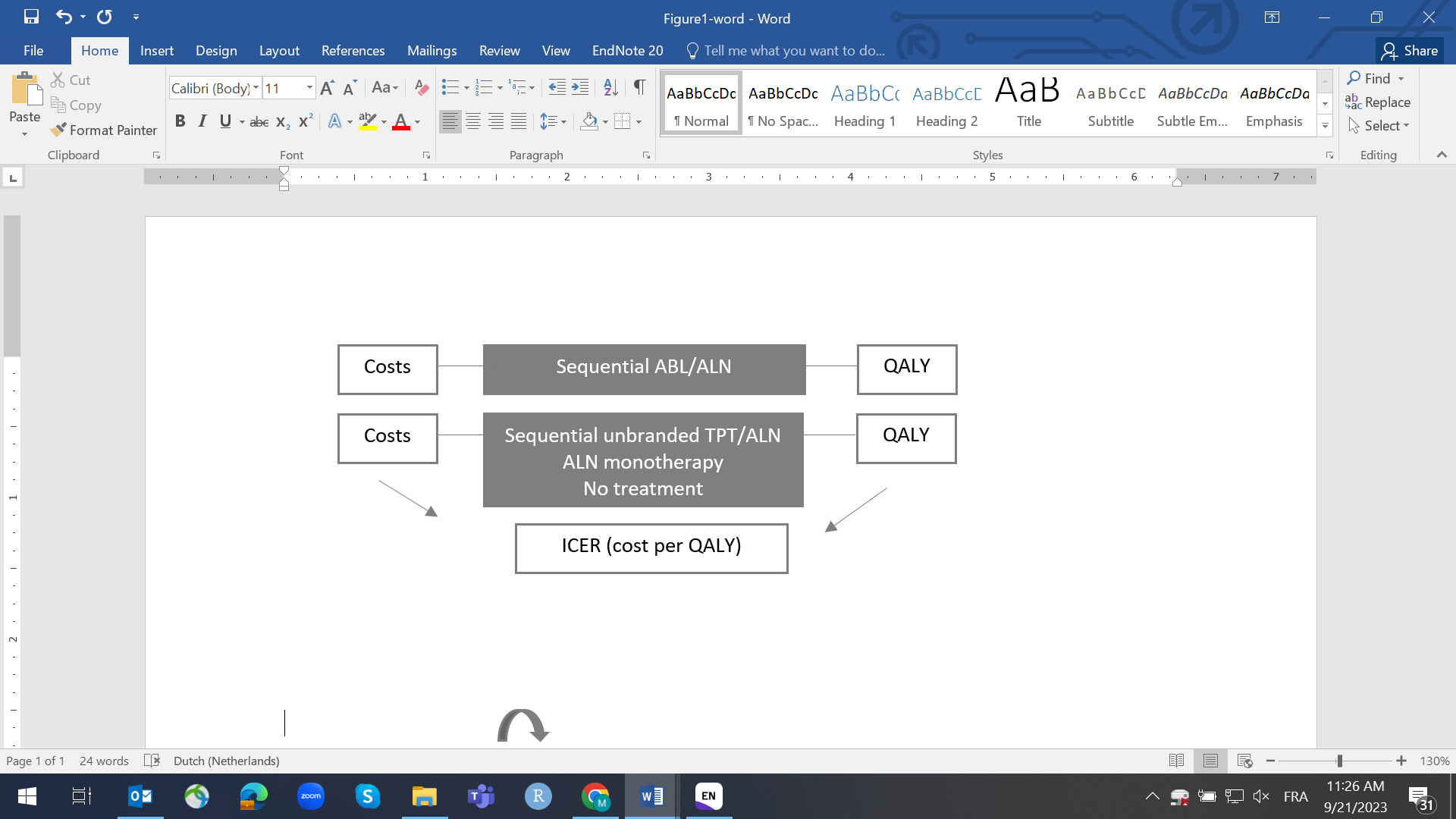

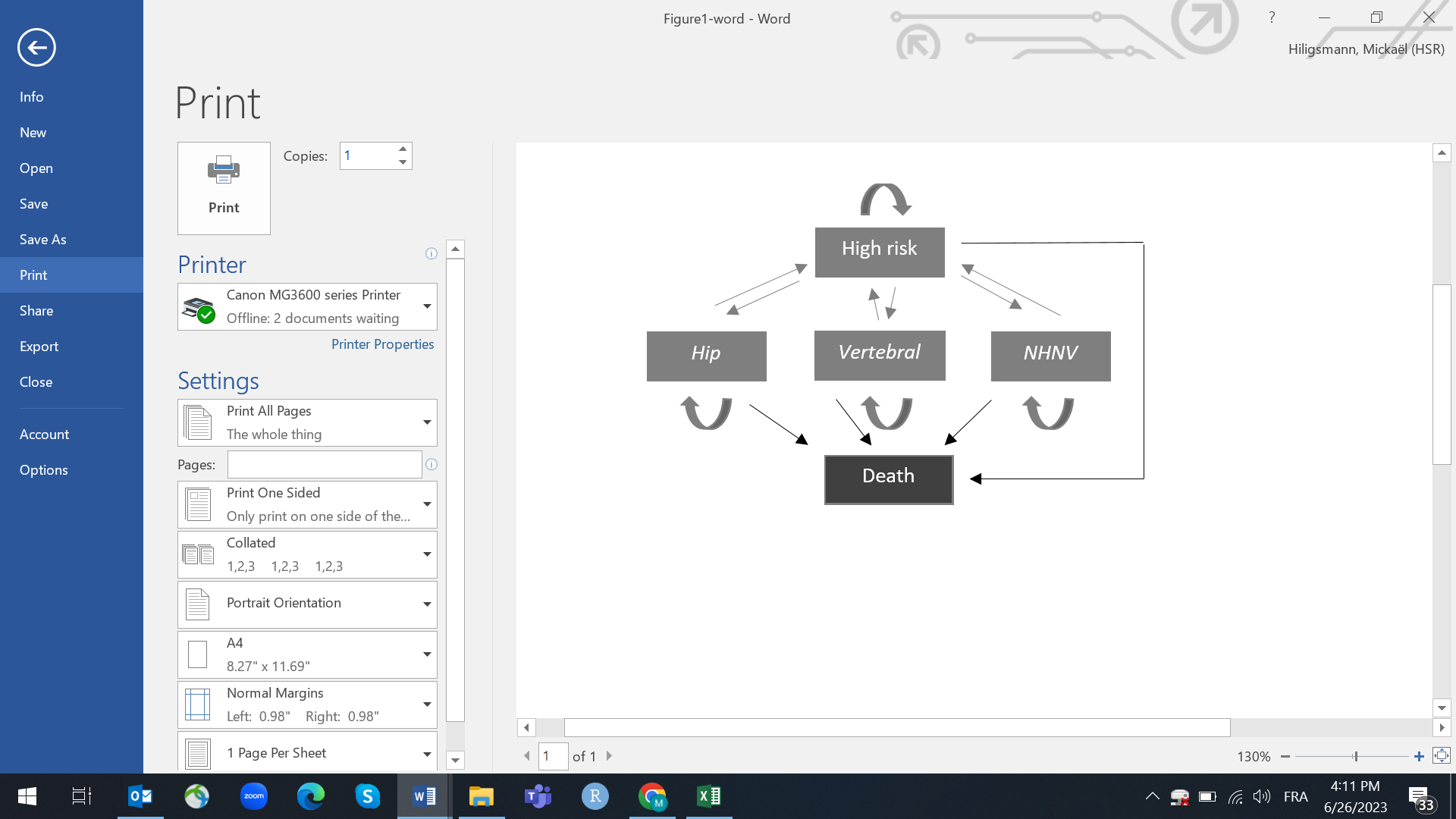


*ABL* abaloparatide, *ALN* alendronate, *Fx* fracture*, ICER* incremental cost-effectiveness ratio*, NHNV* nonhip nonvertebral*, QALY* quality-adjusted life year, *TPTD* teriparatide

The health economics analysis compared sequential ABL/ALN versus sequential unbranded TPTD/ALN, ALN monotherapy, and no treatment in terms of costs and QALYs. Results of the analysis are expressed in ICER. The model health states include “high risk,” “hip fracture,” “vertebral fracture,” “NHNV” and “death.”
