# Supplementary material for: Comparison of the cost-effectiveness of sequential treatment with abaloparatide in US men and women at very high risk of fractures
Source: Aging Clin Exp Res. 2024 Jan 30;36(1):14. doi: 10.1007/s40520-023-02682-7 (PMC10827834; doi:10.1007/s40520-023-02682-7)
Supplement: Supplementary file 2 — Supplementary file2 (DOCX 25 KB) [file 40520_2023_2682_MOESM2_ESM.docx]

# Online Resource 2: Distributions for the probabilistic sensitivity analyses

| Parameter | | Distribution |
| --- | --- | --- |
| Fracture risk | |  |
| Hip fracture, CV fracture, wrist fracture, and other fracture risk | | Beta |
| Relative risk of a prior fracture on future fracture risk (for age range 60 to 70 y) | | Log normal |
| Treatment effects | Log normal | |
| Mortality | |  |
| Excess mortality after fractures | | Log normal |
| Utility | |  |
| Relative risk of the effects of fractures | | Beta |
| Fracture costs | |  |
| First year and subsequent year cost of a fracture | | Normal (SE=20% of the mean) |

*SE* standard error
