# Supplementary material for: Comparison of the cost-effectiveness of sequential treatment with abaloparatide in US men and women at very high risk of fractures
Source: Aging Clin Exp Res. 2024 Jan 30;36(1):14. doi: 10.1007/s40520-023-02682-7 (PMC10827834; doi:10.1007/s40520-023-02682-7)
Supplement: Supplementary file 4 — Supplementary file4 (DOCX 83 KB) [file 40520_2023_2682_MOESM4_ESM.docx]

**Online Resource 4**

**Table S1:** Comparison of the incremental cost-effectiveness ratio (expressed in cost in US$ per QALY gained) of ABL/ALN compared with alternative treatments according to treatment efficacy scenarios in US men at very high risk of fractures (defined as having any recent fracture and BMD T-score ≤−2.5), at different ages

|  | ***Clinical trials efficacy*** | | | ***NMA efficacy*** | | |
| --- | --- | --- | --- | --- | --- | --- |
|  | **ABL/ALN vs no treatment** | **ABL/ALN vs unbranded TPTD/ALN** | **ABL/ALN vs ALN monotherapy** | **ABL/ALN vs no treatment** | **ABL/ALN vs unbranded TPTD/ALN** | **ABL/ALN vs ALN monotherapy** |
| 50 years | 98,771 | Dominant  (incr costs: −8686  incr QALY: 0.034) | 139,530 | 83,637 | Dominant  (incr costs: −7520  incr QALY: 0.019) | 145,537 |
| 55 years | 97,614 | Dominant  (incr costs: −8610  incr QALY: 0.025) | 146,804 | 70,518 | Dominant  (incr costs: −7522  incr QALY: 0.018) | 124,577 |
| 60 years | 55,243 | Dominant  (incr costs: −8809  incr QALY: 0.027) | 94,568 | 40,615 | Dominant  (incr costs: −7884  incr QALY: 0.021) | 85,629 |
| 65 years | 65,202 | Dominant  (incr costs: −9162  incr QALY: 0.026) | 105,453 | 40,679 | Dominant  (incr costs: −7879  incr QALY: 0.023) | 83,481 |
| 70 years | 45,246 | Dominant  (incr costs: −9211  incr QALY: 0.027) | 84,070 | 20,378 | Dominant  (incr costs: −8189  incr QALY: 0.024) | 60,810 |
| 75 years | 25,908 | Dominant  (incr costs: −9397  incr QALY: 0.026) | 67,686 | Dominant  (incr costs: −591  incr QALY: 0.194) | Dominant  (incr costs: −8649  incr QALY: 0.025) | 32,751 |
| 80 years | 25,543 | Dominant  (incr costs: −9249  incr QALY: 0.024) | 68,713 | Dominant  (incr costs: −1378  incr QALY: 0.180) | Dominant  (incr costs: −8581  incr QALY: 0.025) | 29,272 |

**Table S2:** Comparison of the incremental cost-effectiveness ratio (expressed in cost in US$ per QALY gained) of ABL/ALN compared with alternative treatments according to treatment efficacy scenarios in US women at very high risk of fractures (defined as having any recent fracture and BMD T-score ≤−2.5), at different ages

|  | ***Clinical trials efficacy*** | | | ***NMA efficacy*** | | |
| --- | --- | --- | --- | --- | --- | --- |
|  | **ABL/ALN vs no treatment** | **ABL/ALN vs unbranded TPTD/ALN** | **ABL/ALN vs ALN monotherapy** | **ABL/ALN vs no treatment** | **ABL/ALN vs unbranded TPTD/ALN** | **ABL/ALN vs ALN monotherapy** |
| 50 years | 211,106 | Dominant  (incr costs: −7512  incr QALY: 0.018) | 253,015 | 173,561 | Dominant  (incr costs: −6833  incr QALY: 0.011) | 257,286 |
| 55 years | 123,686 | Dominant  (incr costs: −8206  incr QALY: 0.026) | 166,039 | 114,026 | Dominant  (incr costs: −7267  incr QALY: 0.014) | 177,166 |
| 60 years | 137,580 | Dominant  (incr costs: −8245  incr QALY: 0.017) | 184,376 | 113,118 | Dominant  (incr costs: −7139  incr QALY: 0.017) | 187,469 |
| 65 years | 97,161 | Dominant  (incr costs: −8540  incr QALY: 0.024) | 138,395 | 80,867 | Dominant  (incr costs: −7419  incr QALY: 0.017) | 134,682 |
| 70 years | 77,547 | Dominant  (incr costs: −8533  incr QALY: 0.030) | 113,244 | 56,028 | Dominant  (incr costs: −7621  incr QALY: 0.020) | 99,362 |
| 75 years | 53,624 | Dominant  (incr costs: −9225  incr QALY: 0.030) | 92,140 | 29,263 | Dominant  (incr costs: −8112  incr QALY: 0.021) | 73,752 |
| 80 years | 52,837 | Dominant  (incr costs: −9187  incr QALY: 0.026) | 98,505 | 21,156 | Dominant  (incr costs: −8117  incr QALY: 0.023) | 64,421 |

*ABL* abaloparatide, *ALN* alendronate, *incr* incremental, *NMA* network meta-analysis, *QALY,* quality-adjusted life year, *TPTD* teriparatide

**Table S3:** Comparison of the incremental cost-effectiveness ratio (expressed in cost in US$ per QALY gained) of ABL/ALN compared with alternative treatments according to treatment efficacy scenarios in US men at very high risk of fractures (defined as having a recent hip fracture and BMD T-score ≤−2.5), at different ages

|  | ***Clinical trials efficacy*** | | | ***NMA efficacy*** | | |
| --- | --- | --- | --- | --- | --- | --- |
|  | **ABL/ALN vs no treatment** | **ABL/ALN vs unbranded TPTD/ALN** | **ABL/ALN vs ALN monotherapy** | **ABL/ALN vs no treatment** | **ABL/ALN vs unbranded TPTD/ALN** | **ABL/ALN vs ALN monotherapy** |
| 50 years | 56,379 | Dominant  (incr costs: −9716  incr QALY: 0.033) | 91,879 | 38,609 | Dominant  (incr costs: −7993  incr QALY: 0.024) | 88,073 |
| 55 years | 48,305 | Dominant  (incr costs: −9536  incr QALY: 0.031) | 85,588 | 26,158 | Dominant  (incr costs: −8300  incr QALY: 0.018) | 71,133 |
| 60 years | 18,222 | Dominant  (incr costs: −9991  incr QALY: 0.035) | 48,134 | 4348 | Dominant  (incr costs: −8604  incr QALY: 0.026) | 40,002 |
| 65 years | 25,314 | Dominant  (incr costs: −10,222  incr QALY: 0.036) | 58,800 | 4300 | Dominant  (incr costs: −8708  incr QALY: 0.025) | 43,956 |
| 70 years | 7182 | Dominant  (incr costs: −10,465  incr QALY: 0.031) | 38,542 | Dominant  (incr costs: −2898  incr QALY: 0.204) | Dominant  (incr costs: −9091  incr QALY: 0.028) | 18,437 |
| 75 years | Dominant  (incr costs: −1872  incr QALY: 0.186) | Dominant  (incr costs: −10,620  incr QALY: 0.033) | 23,705 | Dominant  (incr costs: −8129  incr QALY: 0.235) | Dominant  (incr costs: −9727  incr QALY: 0.030) | Dominant  (incr costs: −948  incr QALY: 0.167) |
| 80 years | Dominant  (incr costs: −1871  incr QALY: 0.186) | Dominant  (incr costs: −10,416  incr QALY: 0.031) | 23,331 | Dominant  (incr costs: −9070  incr QALY: 0.215) | Dominant  (incr costs: −9591  incr QALY: 0.031) | Dominant  (incr costs: −1753  incr QALY: 0.156) |

*ABL* abaloparatide, *ALN* alendronate, *incr* incremental, *NMA* network meta-analysis, *QALY,* quality-adjusted life year, *TPTD* teriparatide

**Table S4:** Comparison of the incremental cost-effectiveness ratio (expressed in cost in US$ per QALY gained) of ABL/ALN compared with alternative treatments according to treatment efficacy scenarios in US women at very high risk of fractures (defined as having a recent hip fracture and a BMD T-score ≤−2.5), at different ages

|  | ***Clinical trials efficacy*** | | | ***NMA efficacy*** | | |
| --- | --- | --- | --- | --- | --- | --- |
|  | **ABL/ALN vs no treatment** | **ABL/ALN vs unbranded TPTD/ALN** | **ABL/ALN vs ALN monotherapy** | **ABL/ALN vs no treatment** | **ABL/ALN vs unbranded TPTD/ALN** | **ABL/ALN vs ALN monotherapy** |
| 50 years | 141,712 | Dominant  (incr costs: −7982  incr QALY: 0.026) | 186,967 | 130,294 | Dominant  (incr costs: −7099  incr QALY: 0.014) | 189,815 |
| 55 years | 76,366 | Dominant  (incr costs: −8923  incr QALY: 0.033) | 110,795 | 66,127 | Dominant  (incr costs: −7586  incr QALY: 0.020) | 116,045 |
| 60 years | 82,314 | Dominant  (incr costs: −8908  incr QALY: 0.032) | 117,972 | 73,231 | Dominant  (incr costs: −7630  incr QALY: 0.017) | 124,867 |
| 65 years | 57,585 | Dominant  (incr costs: −9354  incr QALY: 0.032) | 90,609 | 41,278 | Dominant  (incr costs: −7973  incr QALY: 0.022) | 86,007 |
| 70 years | 37,831 | Dominant  (incr costs: −9539  incr QALY: 0.037) | 69,769 | 19,557 | Dominant  (incr costs: -8227  incr QALY: 0.025) | 58,738 |
| 75 years | 14,331 | Dominant  (incr costs: −10,538  incr QALY: 0.034) | 48,513 | Dominant  (incr costs: −1427  incr QALY: 0.187) | Dominant  (incr costs: −8898  incr QALY: 0.025) | 28,148 |
| 80 years | 9318 | Dominant  (incr costs: −10,593  incr QALY: 0.026) | 46,341 | Dominant  (incr costs: −3274  incr QALY: 0.178) | Dominant  (incr costs: −9049  incr QALY: 0.024) | 17,990 |

*ABL* abaloparatide, *ALN* alendronate, *BMD* bone mineral density, *incr* incremental, *NMA* network meta-analysis, *QALY,* quality-adjusted life year, *TPTD* teriparatide

**Table S5:** Comparison of the incremental cost-effectiveness ratio (expressed in cost in US$ per QALY gained) of ABL/ALN compared with alternative treatments according to treatment efficacy scenarios in US men at very high risk of fractures (defined as having a recent vertebral fracture and a BMD T-score ≤−2.5), at different ages

|  | ***Clinical trials efficacy*** | | | ***NMA efficacy*** | | |
| --- | --- | --- | --- | --- | --- | --- |
|  | **ABL/ALN vs no treatment** | **ABL/ALN vs unbranded TPTD/ALN** | **ABL/ALN vs ALN monotherapy** | **ABL/ALN vs no treatment** | **ABL/ALN vs unbranded TPTD/ALN** | **ABL/ALN vs ALN monotherapy** |
| 50 years | 29,437 | Dominant  (incr costs: −10,485  incr QALY: 0.036) | 62,210 | 12,735 | Dominant  (incr costs: −8576  incr QALY: 0.024) | 58,547 |
| 55 years | 21,329 | Dominant  (incr costs: −10,278  incr QALY: 0.035) | 55,182 | 2309 | Dominant  (incr costs: −8614  incr QALY: 0.026) | 43,643 |
| 60 years | Dominant  (incr costs: −808  incr QALY: 0.184) | Dominant  (incr costs: −10,936  incr QALY: 0.031) | 23,792 | Dominant  (incr costs: −3608  incr QALY: 0.212) | Dominant  (incr costs: −9107  incr QALY: 0.032) | 14,746 |
| 65 years | 813 | Dominant  (incr costs: −11,377  incr QALY: 0.037) | 30,122 | Dominant  (incr costs: −3566  incr QALY: 0.202) | Dominant  (incr costs: −9360  incr QALY: 0.027) | 17,231 |
| 70 years | Dominant  (incr costs: −2925  incr QALY: 0.183) | Dominant  (incr costs: −11,614  incr QALY: 0.033) | 12,347 | Dominant  (incr costs: −7712  incr QALY: 0.229) | Dominant  (incr costs: −9687  incr QALY: 0.030) | Dominant  (incr costs: −622  incr QALY: 0.168) |
| 75 years | Dominant  (incr costs: −6620  incr QALY: 0.205) | Dominant  (incr costs: −11,755  incr QALY: 0.035) | Dominant  (incr costs: −343  incr QALY: 0.148) | Dominant  (incr costs: −14,255  incr QALY: 0.265) | Dominant  (incr costs: −10,428  incr QALY: 0.038) | Dominant  (incr costs: −5366  incr QALY: 0.191) |
| 80 years | Dominant  (incr costs: −6722  incr QALY: 0.190) | Dominant  (incr costs: −11,707  incr QALY: 0.031) | Dominant  (incr costs: −754  incr QALY: 0.138) | Dominant  (incr costs: −15,220  incr QALY: 0.244) | Dominant  (incr costs: −10,542  incr QALY: 0.033) | Dominant  (incr costs: −6438  incr QALY: 0.173) |

*ABL* abaloparatide, *ALN* alendronate, *BMD* bone mineral density, *incr* incremental, *NMA* network meta-analysis, *QALY,* quality-adjusted life year, *TPTD* teriparatide

**Table S6:** Comparison of the incremental cost-effectiveness ratio (expressed in cost in US$ per QALY gained) of ABL/ALN compared with alternative treatments according to treatment efficacy scenarios in US women at very high risk of fractures (defined as having a recent vertebral fracture and a BMD T-score ≤−2.5), at different ages

|  | ***Clinical trials efficacy*** | | | ***NMA efficacy*** | | |
| --- | --- | --- | --- | --- | --- | --- |
|  | **ABL/ALN vs no treatment** | **ABL/ALN vs unbranded TPTD/ALN** | **ABL/ALN vs ALN monotherapy** | **ABL/ALN vs no treatment** | **ABL/ALN vs unbranded TPTD/ALN** | **ABL/ALN vs ALN monotherapy** |
| 50 years | 156,165 | Dominant  (incr costs: −7786  incr QALY: 0.024) | 200,423 | 143,752 | Dominant  (incr costs: −7047  incr QALY: 0.012) | 219,670 |
| 55 years | 88,785 | Dominant  (incr costs: −8726  incr QALY: 0.033) | 124,909 | 78,230 | Dominant  (incr costs: −7509  incr QALY: 0.017) | 131,741 |
| 60 years | 99,666 | Dominant  (incr costs: −8723  incr QALY: 0.029) | 138,029 | 82,545 | Dominant  (incr costs: −7399  incr QALY: 0.018) | 139,889 |
| 65 years | 73,516 | Dominant  (incr costs: −9013  incr QALY: 0.030) | 109,852 | 55,889 | Dominant  (incr costs: −7735  incr QALY: 0.018) | 105,990 |
| 70 years | 50,670 | Dominant  (incr costs: −9268  incr QALY: 0.031) | 84,256 | 31,287 | Dominant  (incr costs: −8001  incr QALY: 0.021) | 71,186 |
| 75 years | 28,356 | Dominant  (incr costs: −9907  incr QALY: 0.034) | 62,650 | 3881 | Dominant  (incr costs: −8528  incr QALY: 0.026) | 42,951 |
| 80 years | 24,215 | Dominant  (incr costs: −9944  incr QALY: 0.031) | 62,381 | Dominant  (incr costs: −1078  incr QALY: 0.171) | Dominant  (incr costs: -8741  incr QALY: 0.021) | 33,532 |

*ABL* abaloparatide, *ALN* alendronate, *BMD* bone mineral density, *incr* incremental, *NMA* network meta-analysis, *QALY,* quality-adjusted life year, *TPTD* teriparatide

**Table S7:** One-way sensitivity analyses on the incremental cost-effectiveness ratio of ABL/ALN compared with alternative strategies in both men and women with any recent fracture and BMD T-score ≤−2.5, according to efficacy data scenarios

| Men using clinical trials efficacy | ABL/ALN vs no treatment | ABL/ALN vs unbranded TPTD/ALN | ABL/ALN vs ALN monotherapy |
| --- | --- | --- | --- |
| Base case | 45,246 | Dominant | 84,070 |
| Fracture incidence −25% | 78,439 | Dominant | 123,108 |
| Fracture incidence +25% | 20,868 | Dominant | 55,208 |
| Fracture cost −25% | 65,306 | Dominant | 107,762 |
| Fracture cost +25% | 26,524 | Dominant | 65,570 |
| Fracture disutilities −25% | 56,677 | Dominant | 105,779 |
| Fracture disutilities +25% | 38,384 | Dominant | 70,767 |
| Discount rates 0% | 24,747 | Dominant | 53,647 |
| Discount rates 5% | 60,595 | Dominant | 107,007 |
| No excess mortality | 50,208 | Dominant | 99,892 |
| Recent hip fracture | 7182 | Dominant | 38,542 |
| Recent vertebral fracture | Dominant | Dominant | 12,347 |
| ABL cost −20% | 17,885 | Dominant | 48,082 |
| ABL cost +20% | 70,320 | Dominant | 118,807 |
| ABL cost −50% | Dominant | Dominant | Dominant |
| ABL cost +50% | 113,255 | 175,441 | 170,813 |
| Offset time (linear decline 3 y) | 72,912 | Dominant | 137,018 |
| Offset time (2 y + 3 y decline) | 34,788 | Dominant | 67,075 |
| Complete medication adherence | 31,563 | Dominant | 137,158 |
| Men using NMA efficacy | ABL/ALN vs no treatment | ABL/ALN vs unbranded TPTD/ALN | ABL/ALN vs ALN monotherapy |
| Base case | 77,547 | Dominant | 113,244 |
| Fracture incidence −25% | 94,494 | Dominant | 136,971 |
| Fracture incidence +25% | 60,007 | Dominant | 95,478 |
| Fracture cost −25% | 92,148 | Dominant | 131,061 |
| Fracture cost +25% | 59,542 | Dominant | 96,955 |
| Fracture disutilities −25% | 99,629 | Dominant | 153,159 |
| Fracture disutilities +25% | 63,804 | Dominant | 97,076 |
| Discount rates 0% | 48,931 | Dominant | 79,789 |
| Discount rates 5% | 97,197 | Dominant | 140,090 |
| No excess mortality | 85,186 | Dominant | 126,914 |
| Recent hip fracture | 37,831 | Dominant | 69,769 |
| Recent vertebral fracture | 50,670 | Dominant | 84,256 |
| ABL cost −20% | 44,818 | Dominant | 73,427 |
| ABL cost +20% | 105,642 | Dominant | 154,772 |
| ABL cost −50% | Dominant | Dominant | 14,535 |
| ABL cost +50% | 152,650 | 36,082 | 211,344 |
| Offset time (linear decline 3 y) | 105,741 | Dominant | 158,431 |
| Offset time (2 y + 3 y decline) | 59,500 | Dominant | 88,865 |
| Complete medication adherence | 62,698 | Dominant | 172,884 |

| Women using clinical trials efficacy | ABL/ALN vs no treatment | ABL/ALN vs unbranded TPTD/ALN | ABL/ALN vs ALN monotherapy |
| --- | --- | --- | --- |
| Base case | 20,378 | Dominant | 60,810 |
| Fracture incidence −25% | 53,044 | Dominant | 101,758 |
| Fracture incidence +25% | Dominant | Dominant | 33,425 |
| Fracture cost −25% | 38,413 | Dominant | 77,285 |
| Fracture cost +25% | 1392 | Dominant | 41,738 |
| Fracture disutilities −25% | 25,142 | Dominant | 72,792 |
| Fracture disutilities +25% | 16,988 | Dominant | 50,825 |
| Discount rates 0% | 6,007 | Dominant | 35,597 |
| Discount rates 5% | 33,145 | Dominant | 81,527 |
| No excess mortality | 18,371 | Dominant | 68,724 |
| Recent hip fracture | Dominant | Dominant | 18,437 |
| Recent vertebral fracture | Dominant | Dominant | Dominant |
| ABL cost −20% | Dominant | Dominant | 27,503 |
| ABL cost +20% | 42,842 | Dominant | 90,402 |
| ABL cost −50% | Dominant | Dominant | Dominant |
| ABL cost +50% | 76,027 | 64,803 | 134,742 |
| Offset time (linear decline 3 y) | 44,047 | Dominant | 105,234 |
| Offset time (2 y + 3 y decline) | 11,541 | Dominant | 44,138 |
| Complete medication adherence | 10,017 | Dominant | 129,447 |
| Women using NMA efficacy | ABL/ALN vs no treatment | ABL/ALN vs unbranded TPTD/ALN | ABL/ALN vs ALN monotherapy |
| Base case | 56,028 | Dominant | 99,362 |
| Fracture incidence −25% | 93,718 | Dominant | 150,022 |
| Fracture incidence +25% | 32,200 | Dominant | 72,743 |
| Fracture cost −25% | 72,713 | Dominant | 114,894 |
| Fracture cost +25% | 39,004 | Dominant | 85,229 |
| Fracture disutilities −25% | 68,642 | Dominant | 123,962 |
| Fracture disutilities +25% | 47,101 | Dominant | 86,265 |
| Discount rates 0% | 33,066 | Dominant | 67,549 |
| Discount rates 5% | 75,253 | Dominant | 131,298 |
| No excess mortality | 65,843 | Dominant | 121,845 |
| Recent hip fracture | 19,557 | Dominant | 58,738 |
| Recent vertebral fracture | 31,287 | Dominant | 71,186 |
| ABL cost −20% | 27,847 | Dominant | 61,284 |
| ABL cost +20% | 83,435 | Dominant | 137,465 |
| ABL cost −50% | Dominant | Dominant | 7388 |
| ABL cost +50% | 124,761 | 116,077 | 189,087 |
| Offset time (linear decline 3 y) | 88,753 | Dominant | 163,624 |
| Offset time (2 y + 3 y decline) | 46,410 | Dominant | 85,773 |
| Complete medication adherence | 42,590 | Dominant | 183,582 |

*ABL* abaloparatide, *ALN* alendronate, *BMD* bone mineral density, *NMA* network meta-analysis, *TPTD* teriparatide

**Figure S1:** Cost-effectiveness acceptability curves of ABL/ALN versus alternative treatments in US men and women with BMD T-score ≤−2.5 and a recent hip fracture aged 70 years according to treatment efficacy scenario

*ABL* abaloparatide*, ALN* alendronate*, BMD* bone mineral density, *NMA* network meta-analysis, *QALY* quality-adjusted life year*, TPTD* teriparatide

The curves show the probability that each treatment is cost-effective according to decision maker’s willingness to pay.

**Figure S2:** Cost-effectiveness acceptability curves of ABL/ALN versus alternative treatments in US men and women with BMD T-score ≤−2.5 and a recent vertebral fracture aged 70 years according to treatment efficacy scenario

*ABL* abaloparatide*, ALN* alendronate*, BMD* bone mineral density, *NMA* network meta-analysis, *QALY* quality-adjusted life year*, TPTD* teriparatide

The curves show the probability that each treatment is cost-effective according to decision maker’s willingness to pay.

**Figure S3:** Cost-effectiveness acceptability curves of ABL/ALN compared to ALN monotherapy in men and women aged 70 years with a recent fracture and BMD T-score ≤−2.5 according to treatment efficacy scenarios

*ABL* abaloparatide*, ALN* alendronate*, BMD* bone mineral density, *NMA* network meta-analysis, *QALY* quality-adjusted life year

The curves show the probability that each treatment is cost-effective according to decision maker’s willingness to pay.
